# X‐chromosome-wide association study for Alzheimer’s disease

**DOI:** 10.1038/s41380-024-02838-5

**Published:** 2024-12-04

**Authors:** Julie Le Borgne, Lissette Gomez, Sami Heikkinen, Najaf Amin, Shahzad Ahmad, Seung Hoan Choi, Joshua Bis, Benjamin Grenier-Boley, Omar Garcia Rodriguez, Luca Kleineidam, Juan Young, Kumar Parijat Tripathi, Lily Wang, Achintya Varma, Rafael Campos-Martin, Sven van der Lee, Vincent Damotte, Itziar de Rojas, Sagnik Palmal, EADB, GR@ACE, DEGESCO, EADI, GERAD, DemGene, FinnGen, ADGC, CHARGE, Richard Lipton, Eric Reiman, Ann McKee, Philip De Jager, William Bush, Scott Small, Allan Levey, Andrew Saykin, Tatiana Foroud, Marilyn Albert, Bradley Hyman, Ronald Petersen, Steven Younkin, Mary Sano, Thomas Wisniewski, Robert Vassar, Julie Schneider, Victor Henderson, Erik Roberson, Charles DeCarli, Frank LaFerla, James Brewer, Russell Swerdlow, Linda Van Eldik, Kara Hamilton-Nelson, Henry Paulson, Adam Naj, Oscar Lopez, Helena Chui, Paul Crane, Thomas Grabowski, Walter Kukull, Sanjay Asthana, Suzanne Craft, Stephen Strittmatter, Carlos Cruchaga, James Leverenz, Alison Goate, M. Ilyas Kamboh, Peter St George-Hyslop, Otto Valladares, Amanda Kuzma, Laura Cantwell, Matthias Riemenschneider, John Morris, Susan Slifer, Carolina Dalmasso, Atahualpa Castillo, Fahri Küçükali, Oliver Peters, Anja Schneider, Martin Dichgans, Dan Rujescu, Norbert Scherbaum, Jürgen Deckert, Steffi Riedel-Heller, Lucrezia Hausner, Laura Molina-Porcel, Emrah Düzel, Timo Grimmer, Jens Wiltfang, Stefanie Heilmann-Heimbach, Susanne Moebus, Thomas Tegos, Nikolaos Scarmeas, Oriol Dols-Icardo, Fermin Moreno, Jordi Pérez-Tur, María J. Bullido, Pau Pastor, Raquel Sánchez-Valle, Victoria Álvarez, Mercè Boada, Pablo García-González, Raquel Puerta, Pablo Mir, Luis M. Real, Gerard Piñol-Ripoll, Jose María García-Alberca, Jose Luís Royo, Eloy Rodriguez-Rodriguez, Hilkka Soininen, Alexandre de Mendonça, Shima Mehrabian, Latchezar Traykov, Jakub Hort, Martin Vyhnalek, Jesper Qvist Thomassen, Yolande A. L. Pijnenburg, Henne Holstege, John van Swieten, Inez Ramakers, Frans Verhey, Philip Scheltens, Caroline Graff, Goran Papenberg, Vilmantas Giedraitis, Anne Boland, Jean-François Deleuze, Gael Nicolas, Carole Dufouil, Florence Pasquier, Olivier Hanon, Stéphanie Debette, Edna Grünblatt, Julius Popp, Roberta Ghidoni, Daniela Galimberti, Beatrice Arosio, Patrizia Mecocci, Vincenzo Solfrizzi, Lucilla Parnetti, Alessio Squassina, Lucio Tremolizzo, Barbara Borroni, Benedetta Nacmias, Marco Spallazzi, Davide Seripa, Innocenzo Rainero, Antonio Daniele, Paola Bossù, Carlo Masullo, Giacomina Rossi, Frank Jessen, Victoria Fernandez, Patrick Gavin Kehoe, Ruth Frikke-Schmidt, Magda Tsolaki, Pascual Sánchez-Juan, Kristel Sleegers, Martin Ingelsson, Jonathan Haines, Lindsay Farrer, Richard Mayeux, Li-San Wang, Rebecca Sims, Anita DeStefano, Gerard D. Schellenberg, Sudha Seshadri, Philippe Amouyel, Julie Williams, Wiesje van der Flier, Alfredo Ramirez, Margaret Pericak-Vance, Ole A. Andreassen, Cornelia Van Duijn, Mikko Hiltunen, Agustín Ruiz, Josée Dupuis, Eden Martin, Jean-Charles Lambert, Brian Kunkle, Céline Bellenguez

**Affiliations:** 1 Univ. Lille, Inserm, CHU Lille, Institut Pasteur de Lille, LabEx DISTALZ - U1167-RID-AGE Facteurs de Risque et Déterminants Moléculaires des Maladies Liées au Vieillissement, Lille, France; 2 The John P. Hussman Institute for Human Genomics, University of Miami, Miami, FL USA; 3 Institute of Biomedicine, University of Eastern Finland, Kuopio, Finland; 4 Nuffield Department of Population Health Oxford University, Oxford, UK; 5 Department of Epidemiology, Erasmus MC, Rotterdam, The Netherlands; 6 Department of Biostatistics, Boston University School of Public Health, Boston, MA USA; 7 Department of Medicine, Cardiovascular Health Research Unit, University of Washington, Seattle, WA USA; 8 Department of Old Age Psychiatry and Cognitive Disorders, University Hospital Bonn, University of Bonn, Bonn, Germany; 9 German Center for Neurodegenerative Diseases (DZNE), Bonn, Germany; 10 Division of Neurogenetics and Molecular Psychiatry, Department of Psychiatry and Psychotherapy, University of Cologne, Medical Faculty, Cologne, Germany; 11 Department of Public Health Sciences, University of Miami Miller School of Medicine, Miami, FL USA; 12 Alzheimer Center Amsterdam, Department of Neurology, Amsterdam Neuroscience, Vrije Universiteit Amsterdam, Amsterdam UMC, Amsterdam, The Netherlands; 13 Department of Complex Trait Genetics, Center for Neurogenomics and Cognitive Research, Amsterdam Neuroscience, Vrije University, Amsterdam, The Netherlands; 14 Research Center and Memory Clinic, ACE Alzheimer Center Barcelona, Universitat Internacional de Catalunya, Barcelona, Spain; 15 CIBERNED, Network Center for Biomedical Research in Neurodegenerative Diseases, National Institute of Health Carlos III, Madrid, Spain; 16 Department of Neurology, Albert Einstein College of Medicine, New York, NY USA; 17 Neurogenomics Division, Translational Genomics Research Institute, Phoenix, AZ USA; 18 Arizona Alzheimer’s Consortium, Phoenix, AZ USA; 19 Banner Alzheimer’s Institute, Phoenix, AZ USA; 20 Department of Psychiatry, University of Arizona, Phoenix, AZ USA; 21 Department of Neurology, Boston University, Boston, MA USA; 22 Department of Pathology, Boston University, Boston, MA USA; 23 Program in Translational Neuro-Psychiatric Genomics, Institute for the Neurosciences, Department of Neurology & Psychiatry, Brigham and Women’s Hospital and Harvard Medical School, Boston, MA USA; 24 Program in Medical and Population Genetics, Broad Institute, Cambridge, MA USA; 25 Department of Epidemiology and Biostatistics, Case Western Reserve University, Cleveland, OH USA; 26 Taub Institute on Alzheimer’s Disease and the Aging Brain, Department of Neurology, Columbia University, New York, NY USA; 27 Department of Neurology, Columbia University, New York, NY USA; 28 Department of Neurology, Emory University, Atlanta, GA USA; 29 Department of Radiology, Indiana University, Indianapolis, IN USA; 30 Department of Medical and Molecular Genetics, Indiana University, Indianapolis, IN USA; 31 Department of Neurology, Johns Hopkins University, Baltimore, MD USA; 32 Department of Neurology, Massachusetts General Hospital/Harvard Medical School, Boston, MA USA; 33 Department of Neurology, Mayo Clinic, Rochester, MN USA; 34 Department of Neuroscience, Mayo Clinic, Jacksonville, FL USA; 35 Department of Psychiatry, Mount Sinai School of Medicine, New York, NY USA; 36 Center for Cognitive Neurology and Departments of Neurology, New York University, School of Medicine, New York, NY USA; 37 Department of Psychiatry, New York University, New York, NY USA; 38 Cognitive Neurology and Alzheimer’s Disease Center, Northwestern University Feinberg School of Medicine, Chicago, IL USA; 39 Department of Neurology, Northwestern University Feinberg School of Medicine, Chicago, IL USA; 40 Department of Neurological Sciences, Rush University Medical Center, Chicago, IL USA; 41 Rush Alzheimer’s Disease Center, Rush University Medical Center, Chicago, IL USA; 42 Department of Pathology (Neuropathology), Rush University Medical Center, Chicago, IL USA; 43 Department of Epidemiology and Population Health, Stanford University, Stanford, CA USA; 44 Department of Neurology & Neurological Sciences, Stanford University, Stanford, CA USA; 45 Department of Neurology, University of Alabama at Birmingham, Birmingham, AL USA; 46 Department of Neurology, University of California Davis, Sacramento, CA USA; 47 Department of Neurobiology and Behavior, University of California Irvine, Irvine, CA USA; 48 Department of Neurosciences, University of California San Diego, La Jolla, CA USA; 49 University of Kansas Alzheimer’s Disease Center, University of Kansas Medical Center, Kansas City, KS USA; 50 Sanders-Brown Center on Aging and University of Kentucky Alzheimer’s Disease Research Center, Department of Neuroscience, University of Kentucky, Lexington, KY USA; 51 Michigan Alzheimer’s Disease Center, Department of Neurology, University of Michigan, Ann Arbor, MI USA; 52 Penn Neurodegeneration Genomics Center, Department of Pathology and Laboratory Medicine, University of Pennsylvania Perelman School of Medicine, Philadelphia, PA USA; 53 University of Pittsburgh Alzheimer’s Disease Research Center, Pittsburgh, PA USA; 54 Department of Neurology, University of Southern California, Los Angeles, CA USA; 55 Department of Medicine, University of Washington, Seattle, WA USA; 56 Department of Neurology, University of Washington, Seattle, WA USA; 57 Department of Radiology, University of Washington, Seattle, WA USA; 58 Department of Epidemiology, University of Washington, Seattle, WA USA; 59 Geriatric Research, Education and Clinical Center (GRECC), University of Wisconsin, Madison, WI USA; 60 Department of Medicine, University of Wisconsin, Madison, WI USA; 61 Wisconsin Alzheimer’s Disease Research Center, Madison, WI USA; 62 Gerontology and Geriatric Medicine Center on Diabetes, Obesity, and Metabolism, Wake Forest School of Medicine, Winston-Salem, NC USA; 63 Program in Cellular Neuroscience, Neurodegeneration & Repair, Yale University, New Haven, CT USA; 64 Department of Psychiatry and Hope Center Program on Protein Aggregation and Neurodegeneration, Washington University School of Medicine, St. Louis, MO USA; 65 Cleveland Clinic Lou Ruvo Center for Brain Health, Cleveland Clinic, Cleveland, OH USA; 66 Department of Neuroscience, Mount Sinai School of Medicine, New York, NY USA; 67 Department of Human Genetics, University of Pittsburgh, Pittsburgh, PA USA; 68 Department of Medicine (Neurology), Tanz Centre for Research in Neurodegenerative Disease, Temerty Faculty of Medicine, University of Toronto, and University Health Network, Toronto, ON USA; 69 Taub Institute for Research on Alzheimer’s Disease and the Aging Brain, Department of Neurology, Columbia University Irving Medical Center, 630 West 168th Street, New York, NY 10032 USA; 70 Klinik für Psychiatrie und Psychotherapie, Saarbrücken, Germany; 71 Department of Neurology, Washington University, St. Louis, MO USA; 72 Department of Pathology and Immunology, Washington University, St. Louis, MO USA; 73 Estudios en Neurociencias y Sistemas Complejos (ENyS) CONICET-HEC-UNAJ, Buenos Aires, Argentina; 74 Division of Psychological Medicine and Clinical Neuroscience, School of Medicine, Cardiff University, Wales, UK; 75 Complex Genetics of Alzheimer’s Disease Group, VIB Center for Molecular Neurology, VIB, Antwerp, Belgium; 76 Department of Biomedical Sciences, University of Antwerp, Antwerp, Belgium; 77 German Center for Neurodegenerative Diseases (DZNE), Berlin, Germany; 78 Charité—Universitätsmedizin Berlin, Corporate Member of Freie Universität Berlin, Humboldt-Universität zu Berlin, and Berlin Institute of Health, Institute of Psychiatry and Psychotherapy, Hindenburgdamm 30, 12203 Berlin, Germany; 79 Department for Neurodegenerative Diseases and Geriatric Psychiatry, University Hospital Bonn, Venusberg-Campus 1, 53127 Bonn, Germany; 80 Institute for Stroke and Dementia Research (ISD), University Hospital, LMU Munich, Munich, Germany; 81 German Center for Neurodegenerative Diseases (DZNE), Munich, Germany; 82 Munich Cluster for Systems Neurology (SyNergy), Munich, Germany; 83 Martin-Luther-University Halle-Wittenberg, University Clinic and Outpatient Clinic for Psychiatry, Psychotherapy and Psychosomatics, Halle (Saale), Germany; 84 Department of Psychiatry and Psychotherapy, LVR-Klinikum Essen, University of Duisburg-Essen, Germany, Medical Faculty, Duisburg, Germany; 85 Department of Psychiatry, Psychosomatics and Psychotherapy, Center of Mental Health, University Hospital of Würzburg, Würzburg, Germany; 86 Institute of Social Medicine, Occupational Health and Public Health, University of Leipzig, 04103 Leipzig, Germany; 87 Department of Geriatric Psychiatry, Central Institute for Mental Health Mannheim, Faculty Mannheim, University of Heidelberg, Heidelberg, Germany; 88 Alzheimer’s Disease and Other Cognitive Disorders Unit, Neurology Service, Hospital Clínic of Barcelona, Fundació Recerca Clinic Barcelona- Institut d’Investigacions Biomèdiques August Pi i Sunyer (FRCB-IDIBAPS), and University of Barcelona, Barcelona, Spain; 89 Neurological Tissue Bank-Biobank, Hospital Clinic-FRCB-IDIBAPS, Barcelona, Spain; 90 German Center for Neurodegenerative Diseases (DZNE), Magdeburg, Germany; 91 Institute of Cognitive Neurology and Dementia Research (IKND), Otto-von-Guericke University, Magdeburg, Germany; 92 Center for Cognitive Disorders, Department of Psychiatry and Psychotherapy, Technical University of Munich, School of Medicine and Health, Klinikum rechts der Isar, Munich, Germany; 93 Department of Psychiatry and Psychotherapy, University Medical Center Goettingen, Goettingen, Germany; 94 German Center for Neurodegenerative Diseases (DZNE), Goettingen, Germany; 95 Medical Science Department, iBiMED, Aveiro, Portugal; 96 Institute of Human Genetics, University of Bonn, School of Medicine & University Hospital Bonn, Bonn, Germany; 97 Institute for Urban Public Health, University Hospital of University Duisburg-Essen, Essen, Germany; 98 1st Department of Neurology, Medical school, Aristotle University of Thessaloniki, Thessaloniki, Makedonia Greece; 99 Taub Institute for Research in Alzheimer’s Disease and the Aging Brain, The Gertrude H. Sergievsky Center, Depatment of Neurology, Columbia University, New York, NY USA; 100 1st Department of Neurology, Aiginition Hospital, National and Kapodistrian University of Athens, Medical School, Athens, Greece; 101 Sant Pau Memory Unit, Institut de Recerca Sant Pau (IR Sant Pau), Department of Neurology, Hospital de la Santa Creu i Sant Pau, Barcelona, Spain; 102 Department of Neurology, Hospital Universitario Donostia, San Sebastian, Spain; 103 Neurosciences Area, Instituto Biodonostia, San Sebastian, Spain; 104 Unitat de Genètica Molecular, Institut de Biomedicina de València-CSIC, Valencia, Spain; 105 Unidad Mixta de Neurologia Genètica, Instituto de Investigación Sanitaria La Fe, Valencia, Spain; 106 Centro de Biología Molecular Severo Ochoa (UAM-CSIC), Madrid, Spain; 107 Instituto de Investigacion Sanitaria ‘Hospital la Paz’ (IdIPaz), Madrid, Spain; 108 Universidad Autónoma de Madrid, Madrid, Spain; 109 Fundació Docència i Recerca MútuaTerrassa, Terrassa, Barcelona, Spain; 110 Memory Disorders Unit, Department of Neurology, Hospital Universitari Mutua de Terrassa, Terrassa, Barcelona, Spain; 111 Alzheimer’s Disease and Other Cognitive Disorders Unit, Service of Neurology, Hospital Clínic of Barcelona, Institut d’Investigacions Biomèdiques August Pi i Sunyer, University of Barcelona, Barcelona, Spain; 112 Laboratorio de Genética, Hospital Universitario Central de Asturias, Oviedo, Spain; 113 Instituto de Investigación Sanitaria del Principado de Asturias (ISPA), Oviedo, Spain; 114 Unidad de Trastornos del Movimiento, Servicio de Neurología y Neurofisiología, Instituto de Biomedicina de Sevilla (IBiS), Hospital Universitario Virgen del Rocío/CSIC/Universidad de Sevilla, Seville, Spain; 115 Unidad Clínica de Enfermedades Infecciosas y Microbiología, Hospital Universitario de Valme, Sevilla, Spain; 116 Depatamento de Especialidades Quirúrgicas, Bioquímica e Inmunología, Facultad de Medicina, Universidad de Málaga, Málaga, Spain; 117 Unitat Trastorns Cognitius, Hospital Universitari Santa Maria de Lleida, Lleida, Spain; 118 Institut de Recerca Biomedica de Lleida (IRBLLeida), Lleida, Spain; 119 Alzheimer Research Center & Memory Clinic, Andalusian Institute for Neuroscience, Málaga, Spain; 120 Neurology Service, Marqués de Valdecilla University Hospital (University of Cantabria and IDIVAL), Santander, Spain; 121 Institute of Clinical Medicine—Neurology, University of Eastern Finland, Kuopio, Finland; 122 Faculty of Medicine, University of Lisbon, Lisbon, Portugal; 123 Clinic of Neurology, UH “Alexandrovska”, Medical University—Sofia, Sofia, Bulgaria; 124 Memory Clinic, Department of Neurology, Charles University, Second Faculty of Medicine and Motol University Hospital, Praha, Czech Republic; 125 Department of Clinical Biochemistry, Copenhagen University Hospital—Rigshospitalet, Copenhagen, Denmark; 126 Department of Clinical Genetics, VU University Medical Centre, Amsterdam, The Netherlands; 127 Department of Neurology, Erasmus MC, Rotterdam, The Netherlands; 128 Maastricht University, Department of Psychiatry & Neuropsychologie, Alzheimer Center Limburg, Maastricht, The Netherlands; 129 Unit for Hereditary Dementias, Theme Aging, Karolinska University Hospital-Solna, 171 64, Stockholm, Sweden; 130 Aging Research Center, Department of Neurobiology, Care Sciences and Society, Karolinska Institutet and Stockholm University, Stockholm, Sweden; 131 Department of Public Health and Caring Sciences/Geriatrics, Uppsala University, Uppsala, Sweden; 132 Université Paris-Saclay, CEA, Centre National de Recherche en Génomique Humaine, 91057 Evry, France; 133 Univ Rouen Normandie, Normandie Univ, Inserm U1245 and CHU Rouen, Department of Genetics and CNRMAJ, F-76000 Rouen, France; 134 Inserm, Bordeaux Population Health Research Center, UMR 1219, Univ. Bordeaux, ISPED, CIC 1401-EC, Univ. Bordeaux, Bordeaux, France; 135 CHU de Bordeaux, Pole Santé Publique, Bordeaux, France; 136 Univ. Lille, Inserm 1171, CHU Clinical and Research Memory Research Centre (CMRR) of Distalz, Lille, France; 137 Université de Paris, EA 4468, APHP, Hôpital Broca, Paris, France; 138 University Bordeaux, Inserm, Bordeaux Population Health Research Center, Bordeaux, France; 139 Department of Neurology, Bordeaux University Hospital, Bordeaux, France; 140 Department of Child and Adolescent Psychiatry and Psychotherapy, University Hospital of Psychiatry Zurich, University of Zurich, Zurich, Switzerland; 141 Neuroscience Center Zurich, University of Zurich and ETH Zurich, Zurich, Switzerland; 142 Zurich Center for Integrative Human Physiology, University of Zurich, Zurich, Switzerland; 143 Old Age Psychiatry, Department of Psychiatry, Lausanne University Hospital, Lausanne, Switzerland; 144 Department of Geriatric Psychiatry, University Hospital of Psychiatry Zürich, Zürich, Switzerland; 145 Institute for Regenerative Medicine, University of Zürich, Zurich, Switzerland; 146 Molecular Markers Laboratory, IRCCS Istituto Centro San Giovanni di Dio Fatebenefratelli, Brescia, 25125 Italy; 147 Neurodegenerative Diseases Unit, Fondazione IRCCS Ca’ Granda, Ospedale Policlinico, Milan, Italy; 148 Department of Biomedical, Surgical and Dental Sciences, University of Milan, Milan, Italy; 149 Department of Clinical Sciences and Community Health, University of Milan, 20122 Milan, Italy; 150 Geriatric Unit, Fondazione IRCCS Ca’ Granda Ospedale Maggiore Policlinico, 20122 Milan, Italy; 151 Institute of Gerontology and Geriatrics, Department of Medicine and Surgery, University of Perugia, Perugia, Italy; 152 Division of Clinical Geriatrics, Department of Neurobiology, Care Sciences and Society, Karolinska Institutet, Stockholm, Sweden; 153 Interdisciplinary Department of Medicine, Geriatric Medicine and Memory Unit, University of Bari “A. Moro”, Bari, Italy; 154 Centre for Memory Disturbances, Lab of Clinical Neurochemistry, Section of Neurology, University of Perugia, Perugia, Italy; 155 Department of Biomedical Sciences, Section of Neuroscience and Clinical Pharmacology, University of Cagliari, Cagliari, Italy; 156 Neurology Unit, “San Gerardo” Hospital, Monza and University of Milano-Bicocca, Milan, Italy; 157 Department of Clinical and Experimental Sciences, University of Brescia, Brescia, Italy; 158 Cognitive and Behavioural Neurology, Department of Continuity of Care and Frailty, ASST Spedali Civili Brescia, Brescia, Italy; 159 Department of Neuroscience, Psychology, Drug Research and Child Health University of Florence, Florence, Italy; 160 IRCCS Fondazione Don Carlo Gnocchi, Florence, Italy; 161 Department of Medicine and Surgery, Unit of Neurology, University-Hospital of Parma, Parma, Italy; 162 Department of Hematology and Stem Cell Transplant, Vito Fazzi Hospital, Lecce, Italy; 163 Department of Neuroscience “Rita Levi Montalcini”, University of Torino, Torino, Italy; 164 Department of Neuroscience, Università Cattolica del Sacro Cuore, Rome, Italy; 165 Neurology Unit, IRCCS Fondazione Policlinico Universitario A. Gemelli, Rome, Italy; 166 Laboratory of Experimental Neuropsychobiology, IRCCS Santa Lucia Foundation, Rome, Italy; 167 Institute of Neurology, Catholic University of the Sacred Heart, Rome, Italy; 168 Fondazione IRCCS Istituto Neurologico Carlo Besta, Milan, Italy; 169 Department of Psychiatry and Psychotherapy, Faculty of Medicine and University Hospital Cologne, University of Cologne, Cologne, Germany; 170 Cluster of Excellence Cellular Stress Responses in Aging-associated Diseases (CECAD), University of Cologne, Cologne, Germany; 171 Translational Health Sciences, Bristol Medical School, University of Bristol, Bristol, UK; 172 Department of Clinical Medicine, University of Copenhagen, Copenhagen, Denmark; 173 Laboratory of Genetics, Immunology and Human Pathology, Faculty of Science of Tunis, University of Tunis El Manar, 2092 Tunis, Tunisia; 174 Alzheimer’s Centre Reina Sofia-CIEN Foundation-ISCIII, Madrid, Spain; 175 Krembil Brain Institute, University Health Network, Toronto, ON Canada; 176 Tanz Centre for Research in Neurodegenerative Diseases, Departments of Medicine and Laboratory Medicine & Pathobiology, University of Toronto, Toronto, ON Canada; 177 Department of Population and Quantitative Health Sciences and Cleveland Institute for Computational Biology, Case Western Reserve University, Cleveland, OH USA; 178 Department of Biostatistics, Boston University, Boston, MA USA; 179 Department of Epidemiology, Boston University, Boston, MA USA; 180 Department of Medicine (Biomedical Genetics), Boston University, Boston, MA USA; 181 Department of Ophthalmology, Boston University, Boston, MA USA; 182 Gertrude H. Sergievsky Center, Columbia University, New York, NY USA; 183 Department of Pathology and Laboratory Medicine, University of Pennsylvania Perelman School of Medicine, Philadelphia, PA USA; 184 Glenn Biggs Institute for Alzheimer’s and Neurodegenerative Diseases, San Antonio, TX USA; 185 Boston University and the NHLBI’s Framingham Heart Study, Boston, MA USA; 186 Department of Neurology, Boston University School of Medicine, Boston, MA USA; 187 UK Dementia Research Institute, Cardiff University, Cardiff, UK; 188 Department of Psychiatry & Glenn Biggs Institute for Alzheimer’s and Neurodegenerative Diseases, San Antonio, TX USA; 189 Cologne Excellence Cluster on Cellular Stress Responses in Aging-Associated Disease (CECAD), University of Cologne, Cologne, Germany; 190 Dr. John T. Macdonald Foundation Department of Human Genetics, University of Miami, Miami, FL USA; 191 NORMENT Centre, Division of Mental Health and Addiction, Oslo University Hospital, Oslo, Norway; 192 Institute of Clinical Medicine, University of Oslo, Oslo, Norway; 193 Department of Epidemiology, Biostatistics and Occupational Health, McGill University, Montreal, QC Canada; 194 University of Miami Miller School of Medicine, Miami, FL USA

**Keywords:** Genetics, Diseases, Neuroscience

## Abstract

Due to methodological reasons, the X-chromosome has not been featured in the major genome-wide association studies on Alzheimer’s Disease (AD). To address this and better characterize the genetic landscape of AD, we performed an in-depth X-Chromosome-Wide Association Study (XWAS) in 115,841 AD cases or AD proxy cases, including 52,214 clinically-diagnosed AD cases, and 613,671 controls. We considered three approaches to account for the different X-chromosome inactivation (XCI) states in females, i.e. random XCI, skewed XCI, and escape XCI. We did not detect any genome-wide significant signals (P ≤ 5 × 10^−^^8^) but identified seven X-chromosome-wide significant loci (P ≤ 1.6 × 10^−^^6^). The index variants were common for the Xp22.32, *FRMPD4, DMD* and Xq25 loci, and rare for the *WNK3*, *PJA1*, and *DACH2* loci. Overall, this well-powered XWAS found no genetic risk factors for AD on the non-pseudoautosomal region of the X-chromosome, but it identified suggestive signals warranting further investigations.

## Introduction

Alzheimer’s disease (AD) is a progressive neurodegenerative disease and the most common cause of dementia among the elderly. AD is caused by a combination of modifiable and non-modifiable risk factors, including genetics. Currently, more than 80 genetic loci are associated with AD risk, highlighting several underlying biological mechanisms for AD, including APP metabolism, Tau-mediated toxicity, lipid metabolism or immune-related processes [1–6]. Greater understanding of the genetics of AD is essential to improve the characterization of the pathophysiological processes involved in the disease. However, although the genetic landscape of AD has been extensively studied on the autosomes, little is known about the association of the X-chromosome gene variants with AD risk. To date, large-scale genome-wide association studies (GWAS) did not include the X-chromosome due to the need of specific analyses to account for its features.

While women carry two copies of the X-chromosome, men are hemizygous, meaning they have one X and one Y chromosome. To maintain balance around allelic dosage between the sexes, X-chromosome inactivation (XCI) occurs in females. This process is where one X chromosome is transcriptionally silenced during female development [7, 8]. The choice of the silenced copy is most often random (random XCI or r-XCI), but inactivation can also be skewed toward a specific copy (skewed XCI or s-XCI). Such XCI ‘skewness’ can be subsequently acquired during life and has been described to increase with age in adults [9–12]. Importantly, up to one‐third of X‐chromosome genes ‘escape’ inactivation and are expressed from both X‐chromosomes in female cells (escape XCI or e-XCI). However, these tend to be expressed less from the inactive X-chromosome. Notably, all the genes in the pseudoautosomal region (PAR) 1 of the X-chromosome have Y-chromosome homologues and escape inactivation. Additionally, some genes variably escape inactivation: their expression from the inactive X-chromosome differs between individuals or between cells and tissues within an individual [7, 13]. The inactivation process and the distinction between the PAR and non-PAR regions are thus important considerations when performing an X-chromosome-wide association study (XWAS). For all these reasons, the X-chromosome needs to be treated separately from the autosomes in the quality control (QC), the imputation process and the analysis [14, 15], and has usually been excluded from GWAS, including for the large-scale AD ones. Yet, the X-chromosome represents about 5% of the genome in terms of size and number of genes (UCSC Genome Browser, https://genome.ucsc.edu/cgi-bin/hgTracks?db=hg38&chromInfoPage=), and thus the study of AD genetics remains incomplete.

Several X-chromosome genes have been associated with brain imaging phenotypes [16, 17]. Furthermore, the X-chromosome carries, disproportionately for the whole genome, more than 15% of the known genes related to intellectual disabilities [18]. While genes related to intellectual disabilities are considered to modulate early neurodevelopmental stages well before neurodegenerative processes start, they might impact on the development of cognitive abilities and, potentially, on the establishment of cognitive reserve and brain resilience [19]. Additionally, XCI escape or skewness might contribute to observed sex differences reported in AD [7, 20]. Women have a higher risk of developing dementia than men: in the 65–69 and 85–89 age groups, the prevalence is 1.5% and 24.9% respectively for women, compared with 1.1% and 16.3% for men [21, 22]. However, conclusions regarding differences of incidence across men and women are more mixed [23–27]. Indeed, the sex difference in prevalence can be largely explained by a greater longevity of women, although other factors may also be involved, such as a selective survival bias in men, socio-environmental factors, or different AD-related biological mechanisms between sexes [28]. For example, women have a greater tau burden than men [29–36] and the impact of *APOE* variants on the disease risk or on Tau concentration differs between males and females [37, 38]. Additionally, in the general population, women have better memory performance [39–43]; some studies reported a faster decline of the global cognition in women but results were mixed in the literature [39, 41, 43, 44]. However, among people with mild cognitive impairment, cognitive decline is reportedly faster in women than in men [43, 45, 46]. Additionally, women live longer with AD compared to men [47, 48]. Consistent with this, in AD mouse models, having two X-chromosomes was associated with reduced mortality [47]. This advantage conferred by a second X-chromosome could partly relate to the *KDM6A* gene, which escapes inactivation. A variant of the human version of this gene was associated with an increase in this gene’s expression in the brain, and with less cognitive decline in aging and preclinical AD [47]. Finally, in humans, expression/level of other X-linked genes or proteins are reportedly associated with cognitive change or tau pathology in a sex-specific manner [49, 50].

To investigate the impact of X-chromosome genetic variants on AD risk, we conducted an in-depth XWAS on 115,841 AD cases or AD proxy cases and 613,671 controls from the IGAP (International Genomics of Alzheimer’s Project), EADB (European Alzheimer & Dementia Biobank), UK Biobank (UKB) and FinnGen studies (Supplementary Table S1). We considered three approaches to account for the different inactivation states in females, i.e. r-XCI, s-XCI, and e-XCI [15]. In the r-XCI model, males were considered as homozygous females, while they were coded hemizygous in the e-XCI model. In the s-XCI model, a dominance effect was added to the r-XCI model to account for non-random inactivation in females.

## Method

### Samples

The XWAS is based on 115,841 AD or AD-proxy cases (58% females) and 613,671 controls (55% females) of European ancestry from 35 case-control studies, 2 family studies (LOAD and FHS), and 2 biobanks (UKB and FinnGen) (Supplementary Material and Supplementary Table S1). 55,868 of the 115,841 cases were AD-proxy cases from the UKB. Females were considered as AD-proxy cases if they indicated having at least one parent with dementia [51]. For males, only the mother’s status was used to define the proxy status (Supplementary Material). In a sensitivity analysis including only the diagnosed AD cases, a total of 63,838 AD-cases (59% females) and 806,335 controls (55% females) was considered (Supplementary Table S1).

Additionally, we also analyzed levels of the two cerebro-spinal fluid biomarkers Aβ42 and phosphorylated tau (pTau) in 5522 and 5415 EADB-core samples, respectively [52] (Supplementary Table S2). We also considered 2661 samples with cognitive impairment from three population-based longitudinal studies: AgeCode [53], SNAC-K [54] (both included in EADB-core) and 3C [55] (included in EADI) (Supplementary Table S3). Definitions of cognitive impairment are described in the respective references. These individuals are at increased risk for dementia, but their speed of progression is heterogeneous. For each sample, a Mini-Mental State Examination (MMSE) score was obtained at baseline and in 1 to 5 follow-up sessions.

In addition to the classical autosomal QC, an X-chromosome specific QC was performed prior to imputation for each study (Supplementary Material and Supplementary Table S4). We did not analyze the PAR regions due to a lack of variants on most genotyping chips. Related individuals were excluded from UKB samples but were kept in FinnGen, where related individuals’ exclusion accounts for about 40% of the sample size [56].

Thirty-four studies were imputed with the TOPMed [57] panel (N = 112,690) and three studies were imputed with the 1000 Genomes [58] panel (March 2012) (FHS, CHS and RS, N = 10,102, Supplementary Table S4). The FinnGen was imputed with a Finnish reference panel and the UKB with a combination of 1000 Genomes, HRC [59] and UK10K [60] panels.

### Main analyses

#### Association tests

**Fig. 1:**
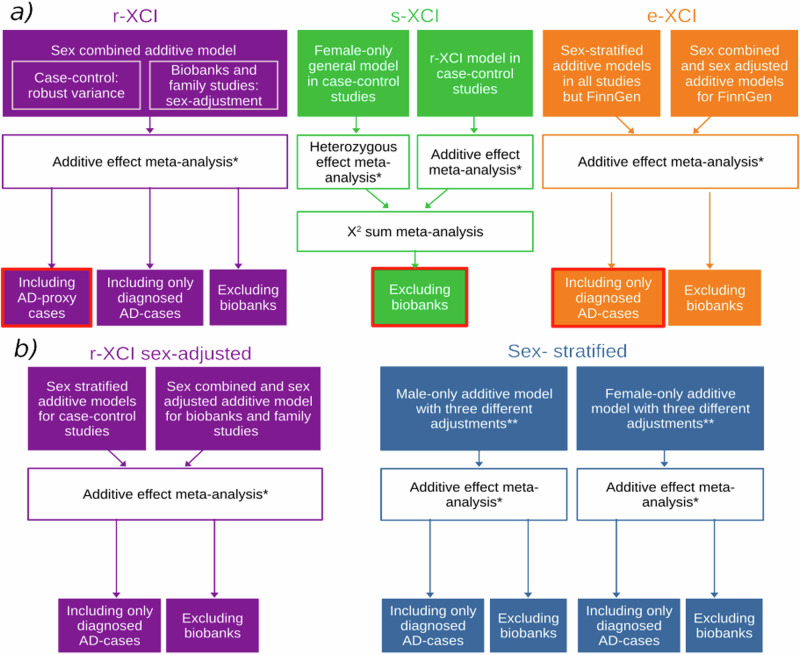
Study design of the AD-XWAS. **a** Main analyses and **b** sensitivity analyses. Box colors indicate the approach: purple, green, orange and blue represent r-XCI, s-XCI, e-XCI and sex-stratified approaches, respectively. Boxes circled in red are the main r-XCI, s-XCI and e-XCI analyses. *Fixed effect meta-analysis with an inverse-variance weighted approach as implemented in METAL [64]. **Sex-stratified models were adjusted on 1) principal components (PCs) and/or the genotyping center; 2) PCs, center and age; 3) PCs, center, age and *APOE*.

Since random X-chromosome inactivation is the most frequent case, we considered the r-XCI approach for our main analysis and the s-XCI and e-XCI approaches for secondary analyses. The approaches are described briefly below, while additional details are provided in the Supplementary Material. An overview of the study design is represented in Fig. 1. For all the models, the analyses were adjusted on the principal components (PCs) and/or the genotyping center if necessary (Supplementary Table S4). Dosage or genotype probabilities were used for all studies but FinnGen, where best guessed genotypes were considered (Supplementary Material).

##### r-XCI approach

The r-XCI approach is equivalent to an additive genetic model, where males are considered as homozygous females. Males’ and females’ genotypes were thus coded: genotype (G) = {0, 2} and G = {0, 1, 2} respectively. The association test was performed for each study in men and women jointly using an additive logistic regression model for case-control studies, a generalized estimating equation (GEE) model for family studies and a logistic mixed model for biobanks. To account for differences in genotypic variance between sexes, we considered a robust estimate of the variance (or Huber-White Sandwich estimator, accounting for heterogeneity of variance within a regression model, Supplementary Material) for case-control studies [61, 62] and an adjustment on sex for family studies and biobanks (Supplementary Table S5). The association test on proxy status in UKB was performed separately for males and females, and a correction factor of 2 was applied to the association statistics (effect sizes and standard errors) of the female-only model (Supplementary Material) [51, 63]. The results were then combined across studies in a fixed effect meta-analysis with an inverse-variance weighted approach with METAL [64].

##### e-XCI approach

Under the e-XCI hypothesis, males’ and females’ genotypes were coded G = {0, 1} and G = {0, 1, 2} respectively. Variant effects were estimated separately in females and in males, except in FinnGen, where the variant effects were estimated directly in both males and females combined with an adjustment on sex (Supplementary Table S5). Results were then combined across studies, males and females with a fixed effect meta-analysis, inverse variance weighted approach using METAL. We did not include AD-proxy in the e-XCI meta-analysis. As males and females are related in family studies, only female results from LOAD and FHS were included in the meta-analysis. The sex-stratified models were adjusted on PCs and/or the genotyping center only, except for two ADGC studies (PFIZER and TGEN2) and the CHARGE studies (FHS, RS and CHS), where models were additionally adjusted on age (Supplementary Table S6 and Supplementary Material).

##### s-XCI approach

For the s-XCI approach, males’ and females’ genotypes were coded G = {0, 2} and G = {0, 1, 2} respectively. A general genotypic model, including both an additive and a dominance variable, was estimated in females from case-control studies to account for non-random inactivation through the dominance variable, which equals 1 in female heterozygotes, and 0 otherwise. The χ^2^ test of the dominance effect was then added to the χ^2^ test of the additive effect estimated under r-XCI, which results in a two degree of freedom (df) test of the association of the variant with AD risk including its potential skewedness [62, 65] (Supplementary Table S5). We did not include family studies and biobanks in the s-XCI approach.

While analyses and QC of the results (see below) were performed with the coding scheme described above, odds-ratio and confidence intervals are provided on the real XCI scale, i.e. G = {0, 1} for males and G = {0, 0.5, 1} for females under r-XCI and s-XCI, but G = {0, 1} for males and G = {0, 1, 2} for females under e-XCI (Supplementary Table S5).

##### Sex-stratified analyses

As the XCI mechanism induces variability across females, one might expect stronger effects in males compared to females; we therefore performed an additional sex-stratified analysis and compared the variant effect sizes in males and females. Proxy cases were not included in this analysis. We combined the results across studies in males and females separately with a fixed effect meta-analysis and inverse-variance weighted approach using METAL [43, 64]. The variant effect sizes of males and females were then compared with a Wald test (Supplementary Material).

#### Quality control of the results and definition of associated loci

A QC of the results was carried out for all the studies. We filtered out variants with at least one missing datum (on effect, standard error, or *p*-value), an absolute effect size greater than 5, or an imputation quality less than 0.3. We also filtered out the variants whose effective allele count (product of the imputation quality and the expected minimum minor allele count between the cases and the controls) was less than 5, and less than 10 for LOAD [66]. For datasets imputed with 1000 G and the UKB, we excluded variants for which the conversion of position or alleles from GRCh37 to GRCh38 was not possible or problematic, and variants with a difference in frequency >0.5 compared with the reference panels TOPMed or 1000 G.

After the meta-analysis, we filtered the variants analyzed in less than 40% of AD cases (considering the effective sample size of females UKB-proxy, which is the raw sample size divided by four [51]), variants with heterogeneity *p*-value < 5 × 10^−8^ and variants where the difference between the maximum frequency and the minimum frequency across studies was higher than 0.4.

Inflation of the test statistics was checked in each study and in the meta-analysis by computing a genomic inflation factor λ with the median approach implemented in the GenABEL 1.8-0 R package [67], on common variants in low LD (r^2^ < 0.2) (Supplementary Material). A signal was considered genome-wide or X-chromosome-wide significant in either approach if associated with AD risk with P ≤ 5 × 10^−8^ or P ≤ 1.6 × 10^−6^. This X-chromosome wide threshold is based on R = 3.12%, the relative number of tests performed on the X-chromosome (n = 888,213, r-XCI approach) versus on the autosomes (n = 27,549,394) in the EADB-core study, the largest dataset imputed with the TOPMed reference panel. As the genome-wide threshold of 5 × 10^−8^ corresponds to the Bonferroni correction for one million tests, we computed the corresponding threshold for the X-chromosome as 0.05 / (R*1,000,000) = 1.6 × 10^−6^.

#### Sensitivity analyses

To account for potential results that we may have missed because of false negatives related to proxy cases or biobanks, we performed sensitivity analyses on the whole X-chromosome excluding these samples for the r-XCI approach and excluding biobank samples for the e-XCI approach (in the first place, proxy cases were not included in the e-XCI analysis and samples from biobanks, including proxy cases, were not included in the s-XCI analysis).

Additionally, several sensitivity analyses of the identified signals were performed. As the robust r-XCI model can generate false positives in the case of differences of frequency between males and females, we performed a sensitivity analysis by adjusting it on sex rather than using a robust variance for the r-XCI signals. The results were obtained by meta-analyzing the sex-stratified models for all case-control studies and UKB, and a sex-combined model adjusted on sex for FinnGen, with males coded as homozygous females for all models (family studies were excluded) (Supplementary Material, Supplementary Table S5). Sensitivity analyses including an adjustment on age and the number of APOE*ε*4 and APOE*ε*2 alleles were also performed for all signals. Results were obtained from the meta-analysis of adjusted sex-stratified models with the adequate coding of males and excluding family studies. Finally, a sensitivity analysis was performed using a stricter imputation quality filter (r^2^ > 0.8).

### Biomarker and cognitive decline analyses

We tested the association of Aβ_42_ and pTau with the genotypes of each common index variant in the EADB-core samples. The analyses were performed separately in 11 batches from 7 European countries (Supplementary Table S2). Following the protocol used by Jansen et al. 2022 [52], we applied a linear regression of the normalized log-transformed levels of Aβ42 and pTau, adjusted for sex, age, assay type (if applicable), and ten PCs, using SNPTEST ‘expected’ method [68]. Genotype probabilities were used for all batches. Additionally to the filters on variants with missing datum, variants with minor allele frequency or MAF < 1% were filtered out, and for batches with less than 250 samples, variants with MAF < 5% were also excluded. We applied this model to both the r-XCI and e-XCI approaches, using a meta-analysis of sex-combined models with an r-XCI coding of the genotypes and a meta-analysis of sex-stratified models with an e-XCI coding of the genotypes, respectively.

Evaluating the link of AD-related X-chromosomal variants with cognitive decline may inform about their contribution to the trajectory of the disease. An association study of cognitive decline was thus performed for the common index variants of the X-chromosome wide significant signals using samples with cognitive impairment from longitudinal studies. Both the r-XCI and e-XCI approaches were considered. The association tests were performed in each study in males and females separately using a linear mixed model. The models tested the association between the normalized MMSE score [69] and the index variant dosage. To account for the random effect between the individuals, we included a “Time” variable, representing the time between the baseline and each follow-up session for each individual. Females were coded G = {0,0.5,1} and G = {0,1,2} for the r-XCI and e-XCI approaches, respectively, and males were coded G = {0,1} in both approaches. Each model was adjusted for age at baseline and four PCs. We also performed sex-combined quadratic models -including the squared effect of “Time”- adjusted on sex, for both r-XCI and e-XCI approaches, as sensitivity analyses [70]. The sex-combined quadratic models were only computed in 3C and AgeCode (the quadratic models did not converge in SNAC-K due to low sample size). We then meta-analyzed together the results of the male-only and female-only linear models of all three studies, and meta-analyzed together the linear and quadratic effects of the sex-combined quadratic model for both approaches.

The significance threshold used for the Aβ_42_, pTau and cognitive decline association test is 4.17 × 10^−3^, which corresponds to the Bonferroni correction for the 4 independent common variants analyzed for 3 phenotypes.

### Colocalization with brain tissue eQTL and pQTL

We performed a genetic colocalization of our hits with brain tissue pQTL and eQTL (protein and expression Quantitative Trait Loci, respectively) for all protein-coding genes within 500 kb of each common index variant, using the “coloc.abf” function from the coloc R-package (version 5.2.3). The brain tissue QTL data were extracted from Wingo et al. 2023 [71], GTex (11 brain tissues) and CommonMind; the last two were processed by the eQTL Catalog (https://www.ebi.ac.uk/eqtl/Data_access/). From the GTex data, we identified 20 genes within 500 kb of the common index variants. We performed the colocalization analyses using the results of the r-XCI or e-XCI analysis where each AD association signal was identified (either the meta-analyses including AD-proxy cases, the diagnosed AD cases meta-analysis or the meta-analysis excluding biobanks). A colocalization between a brain tissue eQTL or pQTL of a gene and an AD-association signal was considered significant when PP4 > 0.75 (posterior probability that both traits are associated and share a single causal variant) [2].

### Differential expression and methylation analyses

To evaluate the biological significance of the genes in the identified loci, we examined differential expression and methylation data in studies comparing AD vs control brains and assessing amyloid plaque burden. We considered 19 genes located at +/- 500 kb of the index variants.

To explore differential expression of our associated loci, we analyzed postmortem brain pathology expression data from individuals of European ancestry using RNA-seq data obtained from temporal lobe and pre-frontal cortex (GEO: GSE44772, GSE33000, GSE118553). Logistic regressions were performed in males and females separately and were adjusted on age at death (age at last visit for clinical AD diagnosis), postmortem interval, RNA integrity, *APOE ε4* status, and first 3 genomic principal components. A significance threshold of 1.32 × 10^−3^ was considered, corresponding to a Bonferroni correction for 19 genes analyzed in two subgroups, that being 38 tests.

Differential methylation of associated loci was assessed by using the DNA Methylation in Aging and Methylation in AD (MIAMI-AD) database (miami-ad.org) [72] which collates results from epigenome-wide association studies in aging and AD. Studies in the database meet two main criteria: 1) having more than 100 total subjects and 2) conducting a genome-wide study of more than 100k CpGs. Details of individual studies can be found on the MIAMI-AD website. Here we queried genes within the associated loci for association with AD neuropathology or dementia, in males and females separately, but also in the combined sample. A significance threshold of 4.39 × 10^−4^ was considered, corresponding to a Bonferroni correction for 19 genes analyzed for 2 phenotypes in 3 subgroups, hence a total 114 tests.

### X-linked intellectual disability (XLID) enrichment analysis

We also tested the enrichment of AD association signals in XLID genes in each approach. We first performed gene-based analyses for each approach (Supplementary Material) and then compared the enrichment of AD association signals in the 156 XLID genes included in the gene-based analysis (out of 164 XLID genes [18]) with the rest of the X-chromosome genes, using MAGMA v1.08 [73].

## Results

### XWAS overview

**Fig. 2:**
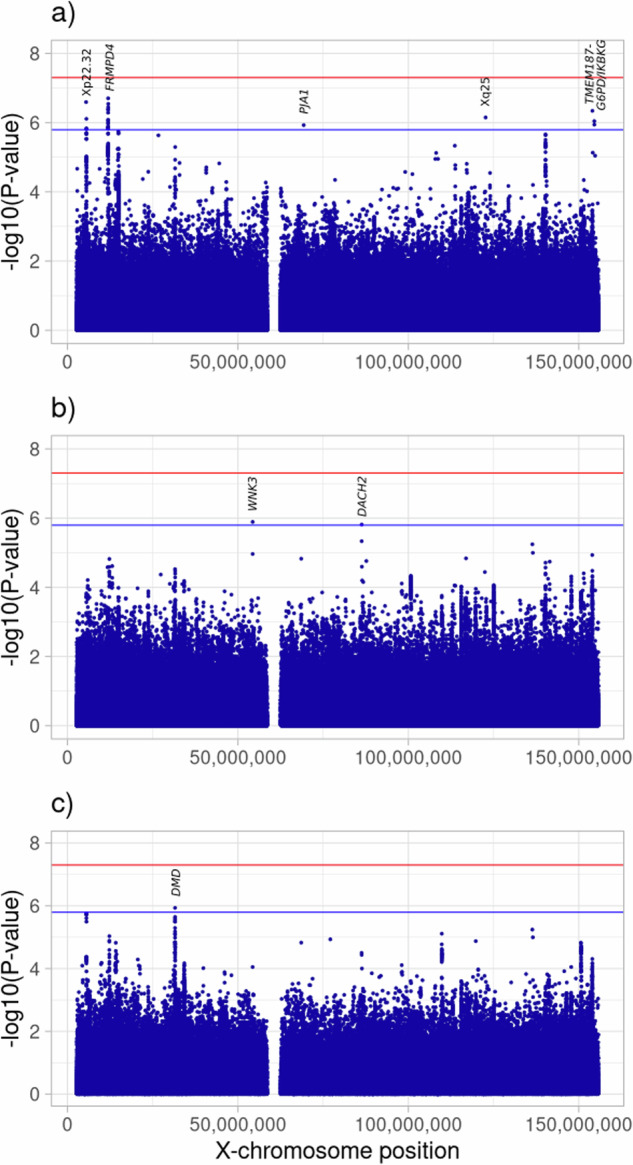
Manhattan plot of the r-XCI approach. Association results of **a** the meta-analysis including AD-proxy cases, **b** the diagnosed AD cases meta-analysis and **c** the meta-analysis excluding biobanks. The red and blue lines represent the genome-wide significant threshold (5 × 10^−8^) and the X-chromosome-wide significant threshold (1.6 × 10^−6^), respectively. The labels show the closest protein-coding gene (according to GENCODE release 45, https://www.gencodegenes.org/human/releases.html) to the index variant of each X-chromosome-wide significant locus.

**Fig. 3:**
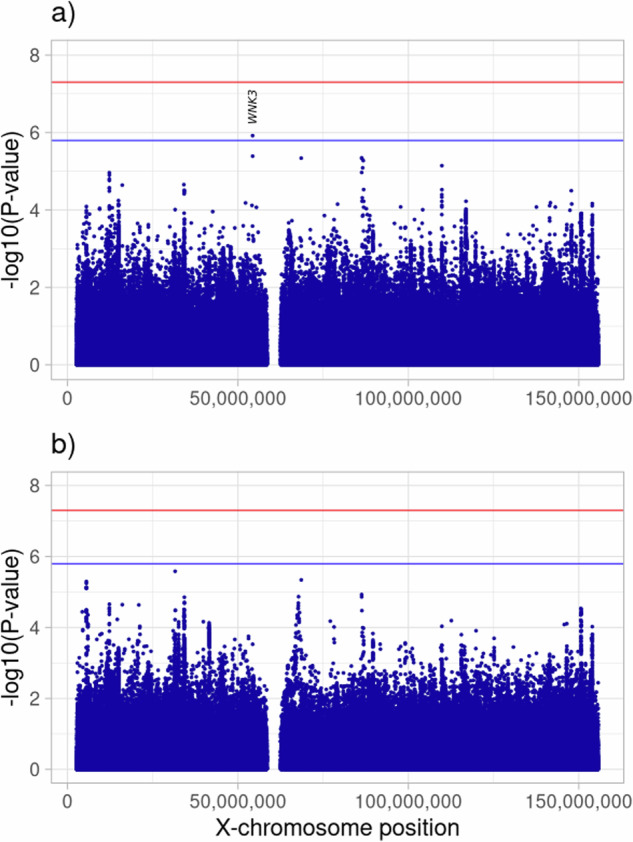
Manhattan plot of the e-XCI approach. Association results of **a** the diagnosed AD-cases meta-analysis and **b** the meta-analysis excluding biobanks. The red and blue lines represent the genome-wide significant threshold (5 × 10^−8^) and the X-chromosome-wide significant threshold (1.6 × 10^−6^), respectively.

**Fig. 4:**
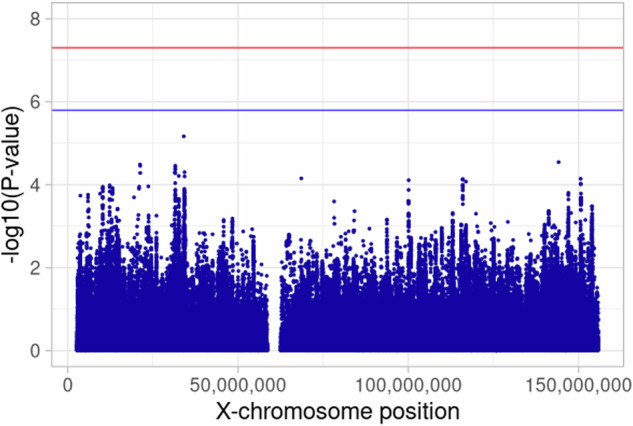
Manhattan plot of the s-XCI approach meta-analysis, which excludes biobanks. The red and blue lines represent the genome-wide significant threshold (5 × 10^−8^) and the X-chromosome-wide significant threshold (1.6 × 10^−6^), respectively.

A total of 666,264, 442,001 and 438,420 variants - including 288,320, 276,902 and 263,169 common variants (MAF ≥ 1%) - were analyzed in the r-XCI, e-XCI and s-XCI approaches, respectively. We observed a minor deviation from expected *p*-values in the r-XCI and e-XCI models (median genomic inflation factor λ = 1.074 and 1.087, respectively) and a deflation in the s-XCI model (median λ = 0.735) (Supplementary Material, Supplementary Figs. S1–S3 and Supplementary Table S7). We did not identify any genome-wide significant signals (P ≤ 5 × 10^−8^) in any of the models (Figs. 2–4). However, five loci exhibited signals that were X-chromosome-wide significant (P ≤ 1.6 × 10^−6^) in the r-XCI approach; the index variants were common for the Xp22.32, *FRMPD4* and Xq25 loci, and rare for the *PJA1* and *TMEM187-G6PD/IKBKG* loci (Fig. 2, Table 1 and Supplementary Table S8). Additionally, a rare variant in the *WNK3* gene was X-chromosome-wide significant in the e-XCI analysis (Fig. 3). No X-chromosome-wide significant signal was found in the s-XCI analysis (Fig. 4). As expected, we observed correlated results between the r-XCI and e-XCI meta-analysis results (Supplementary Table S9).

**Table 1:** Summary of association analysis results with an X-chromosome-wide significant signal.

								Main analysis				Sex-adjusted			
Variant^a^	Model	Meta-analysis	Position^b^	Gene^c^	Inactivation status^d^	Minor/major allele	MAF^e^	OR^f^	95% CI	*p* value	*I*^2^	OR^f^	95% CI	*p* value	*I*^2^
Common variants (MAF > 0.01)															
rs4364769	r-XCI	AD-proxy	5,462,201	*NLGN4X* (Xp22.32)	Variable	T/G	0.12	1.079	1.048–1.110	2.55 × 10^−7^	0	1.074	1.043–1.107	1.52 × 10^−6^	0.0
rs5933929			11,916,372	*FRMPD4*	Inactive	C/A	0.38	0.952	0.935–0.970	1.98 × 10^−7^	0	0.951	0.933–0.969	2.01 × 10^−7^	0.0
rs5972406		excluding biobanks	31,546,147	*DMD*	Inactive	A/G	0.08	1.143	1.083–1.207	1.16 × 10^−6^	0	1.131	1.066–1.200	4.38 × 10^−5^	0.0
rs191195705		AD-proxy	122,643,733	*GRIA3* (Xq25)	NA	A/C	0.11	0.925	0.896–0.954	7.09 × 10^−7^	12.3	0.922	0.894–0.952	6.71 × 10^−7^	10.5
Rare variants (MAF ≤ 0.01)															
rs189139822	r-XCI	AD-Diagnosed	54,307,465	*WNK3*	Inactive	A/G	9.70 × 10^−3^	1.481	1.263–1.735	1.29 × 10^−6^	0	1.475	1.251–1.739	3.67 × 10^−6^	29.7
	e-XCI						9.10 × 10^−3^	1.343	1.192–1.513	1.20 × 10^−6^	15.2				
rs771148434	r-XCI	AD-proxy	69,290,372	*PJA1*	Inactive	G/A	8.00 × 10^−4^	3.107	1.967–4.910	1.18 × 10^−6^	0	3.185	1.986–5.109	1.54 × 10^−6^	0.0
rs1326297223		AD-Diagnosed	86,293,721	*DACH2*	Variable	A/T	2.10 × 10^−3^	2.281	1.629–3.192	1.56 × 10^−6^	0	2.642	1.758–3.970	2.91 × 10^−6^	0.0

*p* values are two-sided raw *p* values derived from a fixed-effect meta-analysis.

*CI* confidence interval, *OR* odds ratio, *MAF* minor allele frequency.

^a^Reference single-nucleotide polymorphism (SNP) (rs) number, according to dbSNP build 153.

^b^GRCh38 assembly.

^c^Nearest protein-coding gene according to GENCODE release 45.

^d^From Tukiainen et al. [13].

^e^Weighted average MAF across all discovery studies.

^f^Approximate OR calculated with respect to the minor allele.

In the sensitivity models including only diagnosed AD-cases or excluding biobank samples, we did not identify any genome-wide significant signals among X-chromosome variants either (Figs. 2 and 3 and Supplementary Figs. S1 and S2). However, we identified an X-chromosome-wide significant signal at a common index variant in the *DMD* locus in the r-XCI meta-analysis excluding biobanks, and at two rare index variants in the *WNK3* and *DACH2* genes in the r-XCI meta-analysis excluding AD-proxy cases (Table 1 and Fig. 2).

In either the male-only or female-only meta-analyses, we did not identify any genome-wide nor X-chromosome-wide significant signals (Supplementary Fig. S4). We also did not observe any genome-wide nor X-chromosome-wide significant difference of effect between males and females for any X-chromosome variants (Supplementary Fig. S4).

### Detailed description of the X-chromosome-wide significant loci

In more details, rs4364769 (MAF = 0.12, OR = 1.079 [1.048–1.110], P = 2.55 × 10^−7^) was identified as the index variant of the Xp22.32 locus in the r-XCI meta-analysis (Table 1 and Supplementary Fig. S5). The odds-ratio estimate of rs4364769 shows some variability across sensitivity analyses but confidence intervals overlap (Supplementary Table S8). The index variant of the Xp22.32 signal is located more than 300 kb from the closest protein coding gene, *NLGN4X* (Neuroligin 4 X-Linked).

The index variant in the *FRMPD4* (FERM and PDZ Domain Containing 4) locus was rs5933929 (MAF = 0.38, OR = 0.952 [0.935–0.970], P = 1.98 × 10^−7^) in the r-XCI meta-analysis (Table 1 and Supplementary Fig. S6). This variant is located in an intron within some transcripts of *FRMPD4*. The odds-ratio of rs5933929 was consistent across sensitivity analyses (Supplementary Table S8).

The common variant rs5972406, located in an intron of the *DMD* dystrophin gene, was X-chromosome-wide significant only in the r-XCI meta-analysis excluding biobanks (MAF = 0.075, OR = 1.143 [1.083–1.207], P = 1.16 × 10^−6^, Table 1 and Supplementary Fig. S7). The odds-ratio estimate was lower in the analysis including AD-proxy cases (OR = 1.075 [1.037–1.113], P = 6.75 × 10^−5^), but confidence intervals overlap (Supplementary Table S8).

rs191195705 was the index variant in the Xq25 signal in the r-XCI meta-analysis (MAF = 0.11, OR = 0.925 [0.896–0.954], P = 7.09 × 10^−7^, Table 1 and Supplementary Fig. S8). Here the males and the UKB-proxy males carried a large part of the observed effect, leading to a lower signal in the sensitivity analyses excluding proxy or biobank cases, or in the female-only compared to the male-only meta-analyses (Supplementary Table S8 and Supplementary Fig. S8). However, the difference of effect between males and females was not significant (P = 0.51, Supplementary Table S8). rs191195705 is over 500 kb from the closest protein coding gene, *GRIA3* (Glutamate Ionotropic Receptor AMPA Type Subunit 3).

Other signals had rare index variants which were not analyzed in the ADGC and CHARGE studies, due to their smaller sample sizes (Supplementary Figs. S9–S12). rs189139822, located in an intron of *WNK3*, was identified in the r-XCI (MAF = 9.70 × 10^−3^, OR = 1.481 [1.263–1.735], P = 1.29 × 10^−6^) and e-XCI (MAF = 9.10 × 10^−3^, OR = 1.343 [1.192–1.513], P = 1.20 × 10^−6^, Supplementary Table S8) meta-analyses excluding AD-proxy cases (Table 1 and Supplementary Fig. S8). The odds-ratio of rs189139822 was consistent across studies and sensitivity analyses (Supplementary Table S8). However, the imputation quality for the variant was low (r^2^ < 0.6) in many studies (Supplementary Fig. S8).

rs771148434, the index variant of the *PJA1* (Praja Ring Finger Ubiquitin Ligase 1) signal, was very rare (MAF = 8.00 × 10^−4^) and analyzed only in the EADB-core and UKB-proxy studies. Detected in the r-XCI meta-analysis (OR = 3.107 [1.967–4.910], P = 1.18 × 10^−6^, Table 1 and Supplementary Fig. S10), this variant was excluded from most of the sensitivity analyses due to its rarity (Supplementary Table S8).

The rs1326297223 index variant is located in an intron of *DACH2*. It was identified in the r-XCI meta-analysis excluding AD-proxy cases (MAF = 2.10 × 10^−3^, OR = 2.281 [1.629–3.192], P = 1.56 × 10^−6^, Table 1 and Supplementary Fig. S11), and its odds-ratio was consistent across studies and sensitivity analyses (Supplementary Table S8).

Three rare variants, all in high LD (r^2^ > 0.8), were associated at the X-chromosome-wide significance threshold with AD-risk in the *TMEM187-G6PD/IKBKG* (Transmembrane Protein 187, Glucose-6-Phosphate Dehydrogenase and Inhibitor Of Nuclear Factor Kappa B Kinase Regulatory Subunit Gamma) locus (Supplementary Table S10). They were only analyzed in the EADB-core and UKB-proxy studies, but the signal was heterogeneous across studies (I^2^ = 56.5 for the index variant rs782044000, Supplementary Fig. S12), and carried mainly by EADB-core. In this study, most carriers were from Italy and Greece, and the signal disappeared when excluding Greek samples (P = 0.43 for rs782044000), or when meta-analyzing the per-country results in EADB-core (P = 0.12 for rs782044000, Supplementary Material). One of the three index variants, rs5030868, is the G6PD Mediterranean mutation, which is much more frequent in the Mediterranean region than in the rest of Europe [74, 75]. We thus considered this signal to be falsely inflated in EADB-core due to this population structure.

### Biomarker and cognitive decline associations

We further investigated the association of the common index variants of the suggestive signals with CSF biomarkers and cognitive decline. We found no significant association (P < 4.17 × 10^−3^) with Aβ_42_ or pTau with any common index variant, whatever the approach (Supplementary Table S11), but the *FRMPD4* index variant rs5933929 was significantly associated with cognitive decline in both the r-XCI and e-XCI approaches (P = 2.75 × 10^−3^ and 3.30 × 10^−3^ respectively), and the direction of effect of the cognitive decline and AD-risk associations were consistent (Supplementary Table S12).

### Colocalization with brain tissue eQTL and pQTL

We did not find any significant colocalization of our AD association signals with any brain tissue eQTL or pQTL signal (Supplementary Table S13).

### Differential expression and methylation

We also examined expression and methylation data of the genes within the suggestive loci.

The genes *FRMPD4* (in males)*, GRIA3* (Xq25, in females)*, TSR2* (in the *WNK3* locus), and *EDA* (in the *PJA1* locus) showed significant differential expression (P < 1.32 × 10^−3^) in temporal lobe tissue (Supplementary Table S14). Expression of *EDA* was also significantly associated with AD status in female pre-frontal cortex tissue. Amyloid plaque burden was found to be significantly associated (P < 4.39 × 10^−4^) with methylation changes in *FRMPD4*, *ARHGAP6* (in the *FRMPD4* locus), and *DMD*.

### XLID gene enrichment

All the suggestive loci contain XLID putative causal genes, except *DACH2*. However, we did not detect an enrichment of the AD association signals in the XLID genes in any of the analyses (P = 0.37, 0.33 and 0.53 in r-XCI, e-XCI and s-XCI approaches, respectively).

## Discussion

We conducted the most comprehensive XWAS on AD to date, including 115,841 AD or AD-proxy cases and 613,671 controls and using three complementary models to account for the complexity related to the X-chromosome. Importantly, 52,214 clinically diagnosed AD cases were included, allowing to assess the impact of proxy-AD or biobank cases on the results. Despite not detecting any genome-wide significant signals regardless of the approach used, seven X-chromosome-wide significant loci passed our post-analysis QC. Index variants were common in four loci; the signal in the *FRMPD4* locus was consistent across the sensitivity analyses, showing strong robustness, while the other signals in Xp22.32 (*NLGN4X*), Xq25 (*GRIA3*) and *DMD* showed some variability. Robustness of the results was more difficult to assess for the rare index variants of the *WNK3*, *PJA1*, and *DACH2* loci.

*FRMPD4* (FERM and PDZ domain containing 4) is mostly expressed in brain tissues (GTex Portal, https://gtexportal.org/), and showed differential expression in male temporal lobe between AD cases and controls. Through its interaction with other proteins, the FRMPD4 protein is involved in the regulation of the morphogenesis and density of dendritic spines, and in the maintenance of excitatory synaptic transmission [76]. *FRMPD4* is an X-linked intellectual disability gene [77] and is associated with low educational attainment [78]. The association of the index variant of the *FRMPD4* locus with cognitive decline could thus be linked to a lower cognitive reserve. The associated variant is in an intron within some transcripts of *FRMPD4* but is also close to the *MSL3* gene, which interacts with *KAT8*, a reported genetic risk factor for AD [2, 79, 80]. In addition, *FRMPD4* is an inactivated gene in females, while *MSL3* escapes inactivation [13].

The signal at the intronic variant within the *DMD* dystrophin gene decreased when including proxy or biobank cases; further analyses are necessary to determine whether this is due to a falsely inflated signal in the clinically diagnosed samples, or to a less specific diagnosis in the proxy and biobank samples. *DMD* is inactivated in females [13], and mutations in the gene can cause Duchenne muscular dystrophy. Some patients suffering from this disease can exhibit cognitive impairment, and a shift towards amyloidogenesis in memory-specific brain regions was found in mice mutated in the *DMD* gene (mdx mouse) compared to wild-type mice [81]. Additionally, the *DMD* rs5927116 variant was reportedly associated with the volume of entorhinal cortex in a small sample (N = 792); however, this signal is 1.4 Mb away from our AD signal and the variants are independent (LD measured by r^2^ = 1.65 × 10^−4^) [82].

The rare variant signals in the *WNK3* and *PJA1* loci are characterized by a low imputation quality or a limited number of clinically diagnosed AD cases analyzed, and further analyses in sequencing data would be necessary to validate those signals. Inhibition of WNK3 is reportedly neuroprotective in stroke [83] and intracerebral hemorrhage [84], but has a deleterious effect on neurons after traumatic brain injury [85]. In the *PJA1* locus, the index variant is located in an enhancer between the *PJA1* and *NALF2* (NALCN Channel Auxiliary Factor 2) genes. Variants in this locus are associated with educational attainment [78], and *PJA1* is expressed in the brain. Another rare variant signal was identified in the *DACH2* gene, which is associated with brain shape (segment 7) [86] and edge-level brain connectivity measures [87].

Identifying putative causal genes in the two other loci, Xp22.32 and Xq25, is more challenging, as the index variants are located more than 300 kb away from the closest protein coding gene, *NLGN4X* and *GRIA3*, respectively. Additionally, those variants are not eQTL/sQTL for any gene according to GTeX Portal. Expression of the *GRIA3* gene in temporal lobe is associated with AD risk in females, and its expression in the dorsolateral prefrontal cortex is reportedly associated with cognitive change in women during aging and AD [47]. However, the rs191195705 index variant of the Xq25 signal is associated with AD risk mainly in males in our analyses (Supplementary Table S8). Regarding the Xp22.32 locus, the rs5916169 variant, located at 127 kb from our index variant, is associated with functional connectivity [16]. However, this variant is not in LD (r^2^ = 0.005) with the AD index variant.

Different X-chromosome-wide significant loci were detected in the main analysis—considering AD-proxy cases— and in the sensitivity ones including only diagnosed AD-cases or excluding biobank samples. A loss of significance was expected in the sensitivity analyses compared to the main analysis due to lower power; similar odds-ratio and overlapping confidence intervals should however be observed for signals mainly driven by AD rather than non-AD dementia. This was the case for the *FRMPD4* locus, and to a lesser extent for the Xp22.32 and Xq25 loci. The loss of a signal in the sensitivity analyses might also be due to purely analytical reasons; for example, the rare index variant of the *PJA1* locus did not pass the filtering criteria in the sensitivity analyses. The identification of the *DMD*, *WNK3* and *DACH2* loci in the sensitivity analyses but not in the main analysis, despite its higher power, might be due to sampling variation, a dilution of the signal in the main analysis linked to the expected higher proportion of non-AD dementia cases among AD-proxy cases or a falsely inflated signal in the sensitivity analyses. Additionally, the correction factor used in the UKB proxy analysis was designed for common variants with low to moderate effect. It might be less appropriate for rare variants with larger effects, such as the index variants of the *WNK3* and *DACH2* loci. Further studies in larger samples will help to delineate the real impact of those loci on AD risk.

Although this study represents a powerful XWAS for AD, we did not find any genome-wide-significant genetic association with AD risk among X-chromosome variants. A recent XWAS on AD identified only one genome-wide significant association in the *SLC9A7* locus [88]. We do not replicate this result at the X-chromosome-wide significance level (OR = 1.023 [1.005–1.042], P = 1.36 × 10^−2^ for the index variant rs2142791, and minimum P in the locus of 5.2 × 10^−5^, in the r-XCI meta-analysis including AD-proxy cases, Supplementary Table S15). The lack of signal overlap between the two studies may be explained in part by a different definition of AD and AD-proxy status, leading to an expected higher proportion of non-AD dementia cases in the other study (Supplementary Material).

Technical or analytical reasons can partly explain the absence of genome-wide significant signals on the X-chromosome, such as: 1) overall lower variant density, 2) lower coverage by genotyping platforms, 3) lower call rate of variants, 4) lower imputation quality, or 5) a lower effective sample size in males on the X-chromosome compared to the autosomes [89]. However, it is also possible that fewer genome-wide significant associations of X-chromosome loci with AD risk exist than on autosomes due to a lower density of functional variants on the X-chromosome. Indeed, Gorlov et al. 2023 [89] found a lower density of variants in both exonic and intronic regions on the X-chromosome compared to autosomes, which they link to a stronger selection against X-chromosome mutations.

In conclusion, this XWAS found no common genetic risk factor for AD on the non-pseudoautosomal region of the X-chromosome but identified suggestive signals with moderate impact on AD risk, which warrant further investigations. In particular, future analyses of sequencing data will help to address some of the technical issues described above, and will allow to study the impact of X-chromosome rare variants or structural variants on AD risk. Additionally, extending XWAS to AD-related phenotypes, such as cognitive decline, AD pathology or AD biomarkers, would further delineate the impact of X-chromosome genetic variations on the processes leading to AD. Lastly, insights into the contribution of the X-chromosome to AD or AD-related phenotypes will be provided by additional studies of the impact of X-chromosome biology beyond genetic variations, for example gene expression or epigenetic alterations, including parental imprinting [49, 90, 91].

## Supplementary information

Supplementary Material

Supplementary Tables

## Data Availability

Summary statistics are available through the European Bioinformatics Institute GWAS Catalog (https://www.ebi.ac.uk/gwas/) under study accessions GCST90449045 to GCST90449052.

## Code availability

We used publicly available software for all analyses, which are listed in the Supplementary Materials with their appropriate citations. We also provide command lines in the Supplementary Materials appendix, and some specific scripts on Zenodo (10.5281/zenodo.14001011).
